# The Surgical Treatment of Severe Endometriosis Positively Affects the Chance of Natural or Assisted Pregnancy Postoperatively

**DOI:** 10.1155/2015/438790

**Published:** 2015-07-12

**Authors:** Erin M. Nesbitt-Hawes, Neil Campbell, Peta E. Maley, Haryun Won, Dona Hooshmand, Amanda Henry, William Ledger, Jason A. Abbott

**Affiliations:** ^1^University of New South Wales, Sydney, Australia; ^2^Royal Hospital for Women, Locked Bag 2000, Barker Street, Randwick, NSW 2031, Australia

## Abstract

*Objective.* To report reproductive outcomes following laparoscopic surgical excision of histologically confirmed r-ASRM stage III-IV endometriosis. *Study Design.* A retrospective cohort study was performed at the Royal Hospital for Women, a university teaching hospital, Sydney, Australia. Women who had fertility-preserving laparoscopic excision of stage III-IV endometriosis from 1997 to 2009 were contacted regarding reproductive outcomes. *Results.* In the study period, 355 women underwent surgery for stage III-IV endometriosis. Follow-up data are available for 253/355 (71%) women. Postoperatively, 142/253 (56%) women attempted to conceive with a conception rate of 104/142 (73%). Confidence intervals for pregnancy for women who were attempting conception (including the nonresponders) range from 104/262 (40%) to 224/262 (85%). Median time to conception was 12 months. No positive prognostic factors for pregnancy were identified on regression analyses. *Conclusions.* These data provide information to women with suspected severe disease preoperatively concerning their likely postoperative fertility outcomes. Ours is a population with severe endometriosis, rather than an infertile population with endometriosis, so caution needs to be applied when applying these data to women with fertility issues alone.

## 1. Introduction

The correlation between endometriosis and infertility is well documented, with monthly fecundity reported as 0.02 to 0.1, compared with couples without endometriosis of 0.07 to 0.2 per month [1, 2]. Despite this reduction in fecundity, a causal relationship has not been proven with theories including distorted anatomy, failure of implantation, hormonal imbalances, and peritoneal dysfunction [3–6]. Laparoscopic excision of endometriosis has been shown to significantly reduce pain and improve quality of life [7–11]; however the effect on fertility is still debated.

The revised American Society of Reproductive Medicine (r-ASRM) staging system for endometriosis does not correlate well with either pain or fertility outcomes [12, 13], although data are available for mild-moderate disease (r-ASRM I-II). Two randomised controlled trials (RCTs) examining surgical treatment of stage I-II endometriosis and reproductive outcomes have contradictory findings [14, 15], with a meta-analysis favouring surgery [16], although controversy continues.

There are no randomised data on the effect of surgery for moderate and severe stage (r-ASRM III-IV) endometriosis on reproductive outcomes. Observational studies examining reproductive outcomes following colorectal resection for severe endometriosis have been reported and provide the best evidence for women with severe disease regarding reproduction [10, 17–21]. The number of patients in these studies is limited (ranging from 46 to 177 women) and there is a low likelihood of a RCT for advanced stage disease given ethical and cost constraints. With data for 253 women, the current study describes the largest cohort of women evaluated for fertility outcomes following laparoscopic excision of stage III-IV endometriosis.

## 2. Materials and Methods

Following approval from the local Human Research Ethics committee (ref.: 09/120), women who underwent laparoscopic surgery for stage III-IV endometriosis from 1997 to 2009 were identified from the Department of Endo-Gynaecology database. Patients having non-fertility-preserving surgery were excluded from the analysis.

Surgical treatment and follow-up were performed in a standardised manner following departmental protocols. Laparoscopy was performed under general anaesthesia using a 4-port approach after establishment of pneumoperitoneum via a Veress needle [22]. A visual inspection of the abdomen and pelvis was undertaken, adhesions were divided as required to normalise anatomy, and endometriosis was resected with monopolar scissors using a retroperitoneal approach [23]. Endometriomas were excised using a stripping technique [24] with the aim of removing all endometriosis. Deeply invasive endometriosis in the cul de sac was removed by sharp dissection with laparoscopic segmental bowel resection undertaken where necessary to completely remove disease [10, 18]. When there was residual disease, likely serosal uterine endometriosis or adenomyosis or incomplete resection, this was noted. Documentation of endometriosis location and r-ASRM stage, intraoperative complications, and associated procedures was undertaken. The surgical technique undertaken during this time was unchanged and outcomes were assessed by time intervals to assess for a potential time-lag bias. Procedures were undertaken by advanced trainees and consultant gynaecological surgeons. Drains were not used in this study [25] and women were discharged from hospital when their pain control was adequate, and they were freely mobilising and had passed a trial of void following indwelling catheter removal. Routine follow-up included a postoperative visit at 4–8 weeks where a physical examination was performed, and enquiries regarding return to activities of daily living and specific questioning regarding bowel, bladder, and sexual function were made. Fertility advice given to women seeking pregnancy following surgery at this department included instruction not to delay attempting conception.

Women identified from the database as having r-ASRM stage III-IV disease were mailed a questionnaire regarding fertility outcomes following their index surgery. Nonrespondents were mailed a second questionnaire if they had not responded in one calendar month and then contacted by telephone if they had not returned this second questionnaire within a further month.

The questionnaire asked patients to self-report on pregnancies and attempts to conceive pre- and postoperatively. Women were asked to report all pregnancies and whether they conceived naturally or through assisted reproductive technology (ART). The pregnancy outcomes were also recorded. Treatments prior to and following the index surgery, including pain relief, hormonal therapies, and other laparoscopies, were also recorded.

Medical records were reviewed and the r-ASRM score was reviewed and confirmed from the operative record. Operation time, intraoperative, and postoperative complications as well as length of stay were recorded. Postdischarge complications and time to return to normal activities of daily life were also documented.

Data were recorded in a purpose-built database using Statistical Package for Social Sciences, version 20 (SPSS, Chicago, IL), with subsequent statistical analyses undertaken with the same software. Levene's test was used to assess data variance. Where appropriate and according to the data distribution, demographic data were compared using Student *t*-, ANOVA, and Chi squared tests. Time-to-pregnancy data were extracted using Kaplan Meier survival analysis. Probability values of less than 5% were considered significant. Cox regression analysis was performed on the data to investigate predictive variables.

## 3. Results

**Figure 1:**
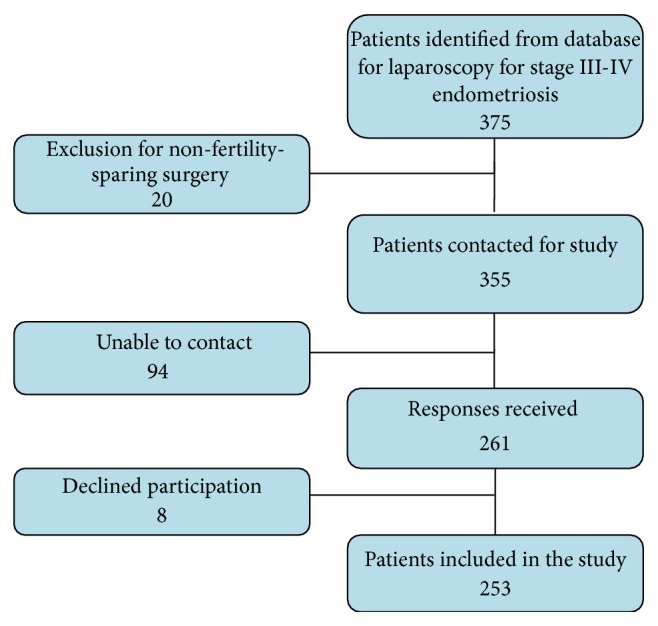
Flow diagram.

From 1997 to 2009, 355 women underwent fertility-preserving laparoscopic surgery by 16 different surgeons for stage III-IV endometriosis. Follow-up was performed to the beginning of 2012. Data were received from 253/355 (71%) women (Figure 1). The median age of respondents was 37 years (range 17–55). Patient demographics are reported in Table 1. Comparing the entire cohort with those who were trying to conceive and those who were successful in conceiving postoperatively, there were no significant differences in age, body mass index (BMI), previous surgery, smoking, indication for index surgery, duration of surgery, previous pregnancy, or length of hospital stay. All women had histologically confirmed endometriosis.

Surgical findings included 155/253 (61%) with stage IV endometriosis; 111/253 (44%) with unilateral or bilateral stripping of endometriomas; 9/253 (3%) women who had laparoscopic segmental bowel resection at index surgery; 35/253 (14%) of women who had suspected adenomyosis or incomplete resection of disease. Reasons for incomplete resection included cervical disease or likely adenomyosis 22/35 (63%); beyond the scope of consent 8/35 (22%); or beyond the skill of staff 5/35 (15%).

Postoperatively, 142/253 (56%) women attempted to conceive, with 104/142 (73%) women achieving at least one pregnancy. In addition, there were five pregnancies in women who were not trying to conceive, for a total of 109 women becoming pregnant. There was no significant difference in fertility for women trying to conceive postoperatively with stage III endometriosis compared to women with stage IV disease 63/91 (69%).

Of the planned pregnancies, 66/104 (63%) were spontaneously conceived and 38/104 (37%) followed ART. Amongst women who attempted to conceive, 38/104 (27%) were unable to achieve a pregnancy either naturally or with ART. Of these, 20/38 (53%) tried natural methods of conception only, 9/38 (24%) tried ART only, and 9/38 (24%) tried both methods. Postoperatively, 45/104 (43%) women had more than one pregnancy. Thirty-eight women who had been pregnant prior to surgery tried to conceive postoperatively. Of these, 26/38 (68%) succeeded in conceiving. All five unplanned pregnancies occurred in women who had a prior pregnancy.

There were 114 women who had attempted to conceive without success prior to surgery. Of these, 95/114 (83%) attempted to conceive postoperatively, and of those women 67/95 (71%) were successful. Twenty women out of 142 (14%) who were trying for a pregnancy postoperatively had previously undergone ART, 18/20 (90%) with 2 or more (up to 12) attempts. Within this group 10/20 (50%) women conceived; 3/10 (30%) conceptions were natural; 7/10 (70%) women conceived with ART.

The outcomes for the first pregnancy (planned or unplanned) following index surgery included term pregnancy in 70/109 (64%); preterm delivery 9/109 (8%) for a live birth rate of 79/109 (72%). The rate of first trimester miscarriage was 23% (25/109). One of 109 (1%) pregnancies resulted in a midtrimester miscarriage and there were 4/109 (4%) terminations of pregnancy. There were no ectopic pregnancies.

**Figure 2:**
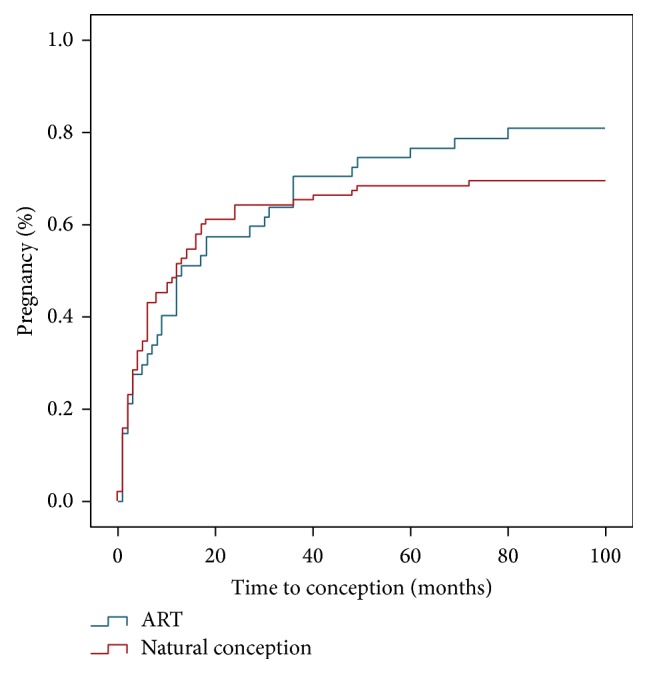
Postoperative pregnancy (women attempting to conceive).

Kaplan Meier survival curve analysis (Figure 2) demonstrates a median time to pregnancy of 12 months following commencement of attempt to conceive for all pregnancies (95% CI 7–17 months). The median length of time to conception was 13 months (95% CI 5–20 months) for those conceiving using ART and 12 months (95% CI 5–19 months) for those who conceived naturally. For women planning to conceive by ART (24/142 (17%)) postindex surgery, 8/24 (33%) had tried ART prior to surgery and 6/24 (25%) had male factor infertility in addition to endometriosis. 10/24 (41%) were recommended to commence ART due to age or other factors. In this group there was 1/24 (4%) spontaneous conception prior to commencing ART and 14/24 (58%) who conceived with IVF.

**Figure 3:**
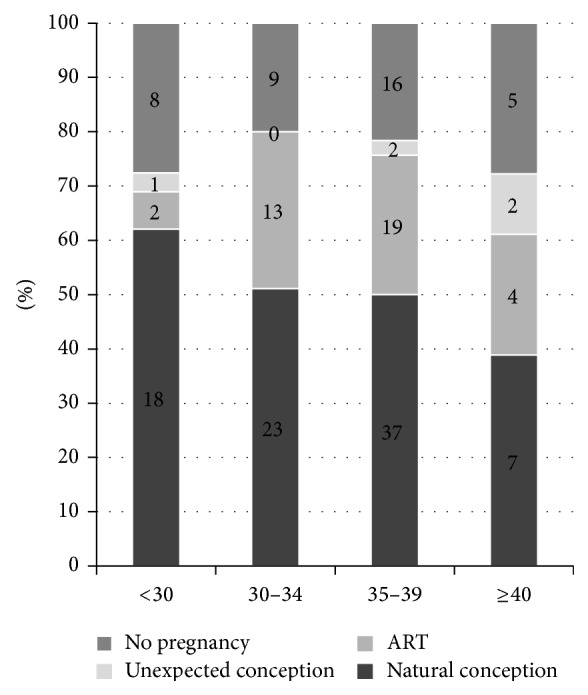
Pregnancy distribution across age bands (women attempting to conceive *n* = 142 and women with unexpected conception *n* = 5).

No positive prognostic factors for conception were identified on regression analysis of the data. In particular, when comparing age bands 17–30, 30–34, 35–39, and 40–55, there was no significant difference (Figure 3). There were no differences in fertility outcomes when time intervals were compared, accounting for the long duration of the study or by surgeon (advanced trainee or consultant). There was also no difference in pregnancy rate for women with a previous pregnancy, those who had used medical treatments prior to their index surgery (the oral contraceptive pill, progestins, or gonadotrophin releasing hormone analogues), or those who had been trying for a pregnancy preoperatively. There were no differences in pregnancy rates for women who had endometriomas resected 64/111 (58%); women having a bowel resection 5/9 (55%); or those with incomplete resection of disease 16/35 (46%) compared with those who did not.

There were 13 recorded complications. These included two major intraoperative complications, one of blood loss >2000 mL requiring a blood transfusion and one unintentional trauma to the bladder repaired laparoscopically. There were four minor intraoperative complications of unintentional entry into the vagina, with one of these requiring a second suturing due to postoperative dehiscence. There were one case of pulmonary oedema, two cases of urinary retention, one urinary tract infection, and two patients who had swelling or bleeding at a laparoscopic port site.

## 4. Discussion

Surgical excision of moderate-to-severe stage endometriosis has been demonstrated to improve women's pain symptoms and quality of life in randomised placebo controlled trials [7, 18, 26]. For fertility outcomes, the largest randomised placebo controlled trial assessed only stage I-II disease and reported an improvement in live birth rate following surgical excision [14]. No RCTs exist for the reproductive outcomes of moderate-to-severe disease and the evidence for this group of women is limited to less robust data [3, 10, 17–20, 27]. Given the ethical and fiscal limitations of undertaking RCTs for advanced disease, it is unlikely that these data will ever be available and we must rely on other sources to fully inform women of what their outcomes are likely to be with severe disease treated surgically.

Many women with endometriosis seek treatment of disease for pain but also desire fertility either immediately following treatment or at some stage in their future [7, 8, 17–19, 27, 28]. The findings of this study indicate that women with moderate-severe stage endometriosis have a good chance of pregnancy following laparoscopic resection of their disease, no matter what their initial presentation was, either pain or fertility, with 34% of our cohort having fertility as one of their reasons for undergoing surgery. Additionally, there was no difference in fertility outcomes for those women having surgery primarily for pain or fertility. Information that may be given to women from this study is that pregnancy is likely to happen spontaneously and the median time to conception is 12 months. These data and the time to pregnancy are in keeping with other data from both early stage disease [28] and more advanced disease that included bowel resection [10, 17–19]. Clearly, additional factors such as male factor infertility must be considered separately as an indicator of need to progress to ART if required.

We recognise the retrospective nature of this study and the inherent recall bias common to this methodology. However, pregnancy is readily defined and women rarely forget details of pregnancy, no matter its outcome; therefore it is likely that the recall of patients in our cohort is correct. A further potential limitation of our data are those lost to follow-up; however our inclusion rate of 71% over a 13-year period provides a robust data set. A confidence interval for pregnancy may also be arrived at given the nonresponse rate of 29%; if all nonresponders failed to conceive, the lower limit for pregnancy would be 104/262 (40%). Conversely, if all succeeded then the upper limit for pregnancy would be 224/262 (85%). A sensitivity analysis on nonresponders demonstrated no differences in age; disease severity or location or presurgical fertility suggesting generalisability is possible. Nonresponse was primarily due to change of address and lack of forwarding details, with only 8 women contacted refusing participation in the study.

Increasing age is associated with declining fertility and previous work reports it as a significant factor for fertility following resection of moderate-to-severe endometriosis [29–31]. The results from our study that report no difference in pregnancy rates based in age group are somewhat surprising, and a type I error with only 16 women in the cohort of women greater than 40 years of age trying to conceive may be contributory. The mean age for women trying for pregnancy in this cohort was 37 and was not different from the mean age of women achieving pregnancy at 36, with the range in this group to 48 providing valuable preoperative information to women suspected with severe disease regarding likely chances of pregnancy postoperatively.

A further factor that may be affected by a type I error may be the finding of adenomyosis or incomplete resection in only 14% of our population. The finding of adenomyosis has been reported to decrease fertility rates when present [17, 19, 20, 29] including women having bowel resection. With only 8.5% of women in our study having adenomyosis, this finding should be treated with caution when counselling women, since larger studies have suggested an effect on fertility.

These data also provide information on the likely mode and time to conception. Despite the severity of disease, 63% of women who became pregnant conceived naturally with the rate of first trimester loss in keeping with reported data [32, 33]. This rate of natural conception is marginally above that reported from other works reporting pregnancy following resection of advanced disease that ranges from 45 to 58% [10, 17, 19, 31]. Possible reasons for this may be that, for women with both infertility and endometriosis, there is often time pressure to conceive quickly after surgery and women and their treating clinicians may view a trial of natural conception postoperatively as a waste of time, particularly if there had been previously failed ART [27, 28]. The approach from the surgeons in this study was not to undertake ART immediately postoperatively unless indicated, but to try for natural conception. Our results suggest that indeed a trial of natural conception is not time wasted, and there is a high chance of spontaneous conception even following prior attempts at ART in the postoperative period. A time limit of 6–12 months for natural conception seems prudent, with factors such as age and other causes of subfertility such as male factor taken into consideration. Similar outcomes have been previously reported from smaller cohorts [27, 34, 35].

Prior pregnancy did not increase postoperative conception in this study. Possible reasons for this may include previous pregnancies occurring before the establishment of severe endometriosis that has been demonstrated to develop from mild forms over a six-month period [7]. This study was not specific to women with endometriosis and infertility that may also be contributory, since women with pain and endometriosis may not have infertility as an issue and 65% of our cohort had pain as their only indication for surgery. The severity of disease may be a contributor to fertility with the Endometriosis Fertility Index (EFI) established as a means of assessing likely fertility outcomes for infertile women [36] and women without infertility primarily [31]. The data from this study show a high pregnancy rate naturally in women with severe endometriosis who may undergo ovarian stripping for endometriomas or bowel resection and may be used to further counsel regarding pregnancy outcomes when surgery is performed by or under the guidance of expert endometriosis surgeons.

Following surgery five women not trying to conceive became pregnant with one resulting termination. All these women had previously had a pregnancy and due to endometriosis may see themselves as infertile not requiring contraception. Given the results from our study following resection of stage III-IV disease, it would be prudent to recommend contraception to women who do not desire a pregnancy or warn of the possibilities of an unplanned conception after surgery.

The data from this study suggests that surgery performed by or under the guidance of expert surgeons for women with stage III and IV endometriosis yields good pregnancy outcomes. Women may be reassured that pregnancy following excision surgery is common and likely to occur in the first year of trying. These data indicate that women with advanced stages of endometriosis treated surgically may expect reasonable likelihood of fertility.

## Condensation

Women who had surgery to remove stage III-IV endometriosis and subsequently tried to conceive had a 73% chance of pregnancy, the majority within 12 months of index surgery.

## Figures and Tables

**Table 1:** Demographic data.

	All participants (*n* = 253)	Trying to conceive post-op (*n* = 142)	Pregnancy post-op (*n* = 109)
Age (median and range)	37 (17–55)	37 (26–50)	36 (26–48)
BMI (median and IQR)	24 (21–28)	24 (21–28)	24 (21–28)
Previous laparoscopic surgery	149 (59%)	83 (59%)	58 (53%)
Smoking			
Yes	111 (44%)	60 (42%)	46 (42%)
Indication for surgery			
Pain	164 (65%)	58 (41%)	48 (44%)
Fertility	27 (11%)	27 (19%)	24 (22%)
Both	58 (23%)	55 (39%)	35 (32%)
Not stated	4 (1%)	2 (1%)	2 (2%)
Prior pregnancy	79 (31%)	38 (27%)	30 (28%)
Trying to conceive pre-op	114 (45%)	95 (67%)	67 (62%)
Trial ART pre-op	20 (8%)	17 (12%)	10 (9%)
Duration of surgery mins (median and IQR)	120 (90–145)	120 (90–150)	120 (90–140)
r-ASRM stage			
III	98 (39%)	51 (36%)	42 (39%)
IV	155 (61%)	91 (64%)	67 (61%)
Length of stay hours (median and IQR)	44 (29–52)	45 (29–51)	42 (28–50)

SD: standard deviation; IQR: interquartile range; ART: assisted reproductive technology; r-ASRM: revised American Society of Reproductive Medicine.
